# The STAMPEDE2 Trial: a Site Survey of Current Patterns of Care, Access to Imaging and Treatment of Metastatic Prostate Cancer

**DOI:** 10.1016/j.clon.2023.07.009

**Published:** 2023-07-22

**Authors:** H. Abdel-Aty, L. O’Shea, C. Amos, L.C. Brown, E. Grist, G. Attard, N. Clarke, W. Cross, C. Parker, M. Parmar, N. Vas As, N. James

**Affiliations:** * Division of Radiotherapy and Imaging, The Institute of Cancer Research, London, UK; † Department of Radiotherapy, The Royal Marsden Hospital NHS Foundation Trust, London, UK; ‡ The Institute of Clinical Trials & Methodology, Medical Research Council Clinical Trials Unit at UCL, London, UK; § Cancer Institute, University College London, London, UK; ¶ Department of Urology, The Christie and Salford Royal NHS Foundation Trusts, Manchester, UK; ‖ Department of Urology, St James’s University Hospital, Leeds, UK

**Keywords:** 177lutetium-PSMA-617, metastatic hormone-sensitive prostate cancer, niraparib, positron emission tomography/computerised tomography, prostate-specific membrane antigen, stereotactic ablative body radiotherapy

## Abstract

**Aims:**

The forthcoming STAMPEDE2 trial has three comparisons in metastatic hormone-sensitive prostate cancer. We aim to determine clinical practices among STAMPEDE trial investigators for access to imaging and therapeutic choices and explore their interest in participation in STAMPEDE2.

**Materials and methods:**

The survey was developed and distributed online to 120 UK STAMPEDE trial sites. Recipients were invited to complete the survey between 16 and 30 May 2022. The survey consisted of 30 questions in five sections on access to stereotactic ablative body radiotherapy (SABR), 177lutetium-prostate-specific membrane antigen-617 (^177^Lu-PSMA-617), choice of systemic therapies and use of positron emission tomography/computerised tomography and whole-body magnetic resonance imaging.

**Results:**

From 58/120 (48%) sites, 64 respondents completed the survey: 55/64 (86%) respondents were interested to participate in SABR, 44/64 (69%) in ^177^Lu-PSMA-617 and 56/64 (87.5%) in niraparib with abiraterone comparisons; 45/64 (70%) respondents had access to bone, spine and lymph node metastases SABR delivery and 7/64 (11%) to ^177^Lu-PSMA-617. In addition to androgen deprivation therapy, 60/64 (94%) respondents used androgen receptor signalling inhibitors and 46/64 (72%) used docetaxel; 29/64 (45%) respondents would consider triplet therapy with androgen deprivation therapy, androgen receptor signalling inhibitors and docetaxel. Positron emission tomography/computerised tomography was available to 62/64 (97%) respondents and requested by 45/64 (70%) respondents for disease uncertainty on conventional imaging and 39/64 (61%) at disease relapse. Whole-body magnetic resonance imaging was available to 24/64 (38%) respondents and requested by 13/64 (20%) respondents in highly selected patients. In low-volume disease, 38/64 (59%) respondents requested scans at baseline and disease relapse. In high-volume disease, 29/64 (45%) respondents requested scans at baseline, best response (at prostate-specific antigen nadir) and disease relapse; 54/64 (84%) respondents requested computerised tomography and bone scan for best response assessment.

**Conclusion:**

There is noteworthy disparity in clinical practice across current study sites, however most have expressed an interest in participation in the forthcoming STAMPEDE2 trial.

## Introduction

Prostate cancer causes around 12 000 deaths per year in the UK [1]. The STAMPEDE platform trial (ISRCTN78818544) is an innovative multi-arm multi-stage platform trial that has tested 10 different treatments in advanced prostate cancer, hypothesising improved outcomes with upstream treatment intensification. To date, three treatments added to androgen deprivation therapy (ADT) have improved outcomes: docetaxel, abiraterone acetate and prostate radiotherapy in low-burden metastatic disease [2–6] and have become standard of care in international guidelines [7,8]. STAMPEDE2 is a new platform trial continuing on from STAMPEDE and testing three new treatments with the ability to add further treatments in the future.

Radiation-based targeted therapies for metastatic prostate cancer have been of increasing interest. Metastasis-directed therapy with stereotactic ablative body radiotherapy (SABR) in metachronous oligometastatic disease has been shown to delay recurrence in prospective trials [9–13]. No randomised data exist in synchronous metastatic disease.

Comparably, in heavily pre-treated castrate-resistant prostate cancer (CRPC), two randomised trials showed that 177lutetium-prostate-specific membrane antigen-617 (^177^Lu-PSMA-617) improved progression-free survival [14,15] with results from the VISION trial [14] leading to the US Food and Drug Administration approval of ^177^Lu-PSMA-617 and subsequently its wider availability [16].

In addition to radiation-based therapies, molecular targeted therapies with poly (ADP-ribose) polymerase inhibitors (PARPi) in combination with androgen-receptor signalling inhibitors (ARSI) have been investigated in first-line CRPC. Phase III randomised trials recently reported on preferential improved survival in men with homologous recombination repair deficiency and breast cancer gene (BRCA) mutation subgroups [17–19].

The STAMPEDE2 trial aims to investigate these treatments in three new comparisons in men with metastatic hormone-sensitive prostate cancer (mHSPC). Here, we report on results from the STAMPEDE2 trial site survey conducted to explore the interest and technical capacities of STAMPEDE investigators to participate in the STAMPEDE2 trial and determine the patterns of current clinical practice for imaging and therapeutic choices.

## Materials and methods

The site survey was designed by the STAMPEDE2 trial team in April 2022. The aims of the survey were to inform the design of the forthcoming STAMPEDE2 trial design and determine consensus on current practices in the UK reflected by the multiplicity of the STAMPEDE trial participating sites. The survey included a summary and rationale of the STAMPEDE2 trial design with three new comparisons in men with mHSPC, investigating SABR (comparison S), ^177^Lu-PSMA-617 (comparison P) and niraparib (PARPi) with abiraterone acetate plus prednisolone (abiraterone; comparison N). The survey constituted 30 questions in five sections: general questions, questions on access to novel imaging facilities, questions on use of systemic therapies at the treating site, questions on access to SABR delivery and questions on access to ^177^Lu-PSMA-617 (see Supplementary Appendix A). Multiple responses were permitted for selected questions. The survey was conducted using the online platform survey monkey (http://www.surveymonkey.co.uk) and was distributed via an e-mail link from the Medical Research Council Clinical Trials Unit (MRC CTU) to the 120 UK-based sites participating in the STAMPEDE trial (ISRCTN78818544). Principal investigators and/or first recipients of the survey were invited to complete the survey. The survey was active online between 16 and 30 May 2022. Descriptive analysis was utilised using Stata statistical software version 17.0 (Stata Corporation, College Station, TX, USA).

## Results

During the 2-week period of the survey being active, 64 respondents completed the survey from 58 of the 120 (48%) STAMPEDE trial participating sites: 55/64 (86%) respondents were interested to participate in comparison S, 44/64 (69%) respondents were interested in comparison P and 56/64 (87.5%) respondents were interested in comparison N; 62/64 (97%) respondents had access to positron emission tomography/computerised tomography (PET/CT) scans. Of those, 35/62 (56%) had access to PET/CT scans at their treating centre, 23/62 (36%) had access at a neighbouring treating centre and 4/62 (6%) had access at a distance centre with a long referral pathway. Eleven of 64 (17%) respondents did not have direct access to PET/CT scans. Of those, 2/64 (3%) foresaw direct access at their treating centre in less than 12 months, 2/64 (3%) foresaw direct access in 1e3 years 1/64 (1.5%) foresaw direct access in more than 3 years and 6/64 (9%) were unsure or had no planned direct access to PET/CT scans. The types of PET/CT scans to which respondents had access to are summarised in Table 1.

**Fig 1:**
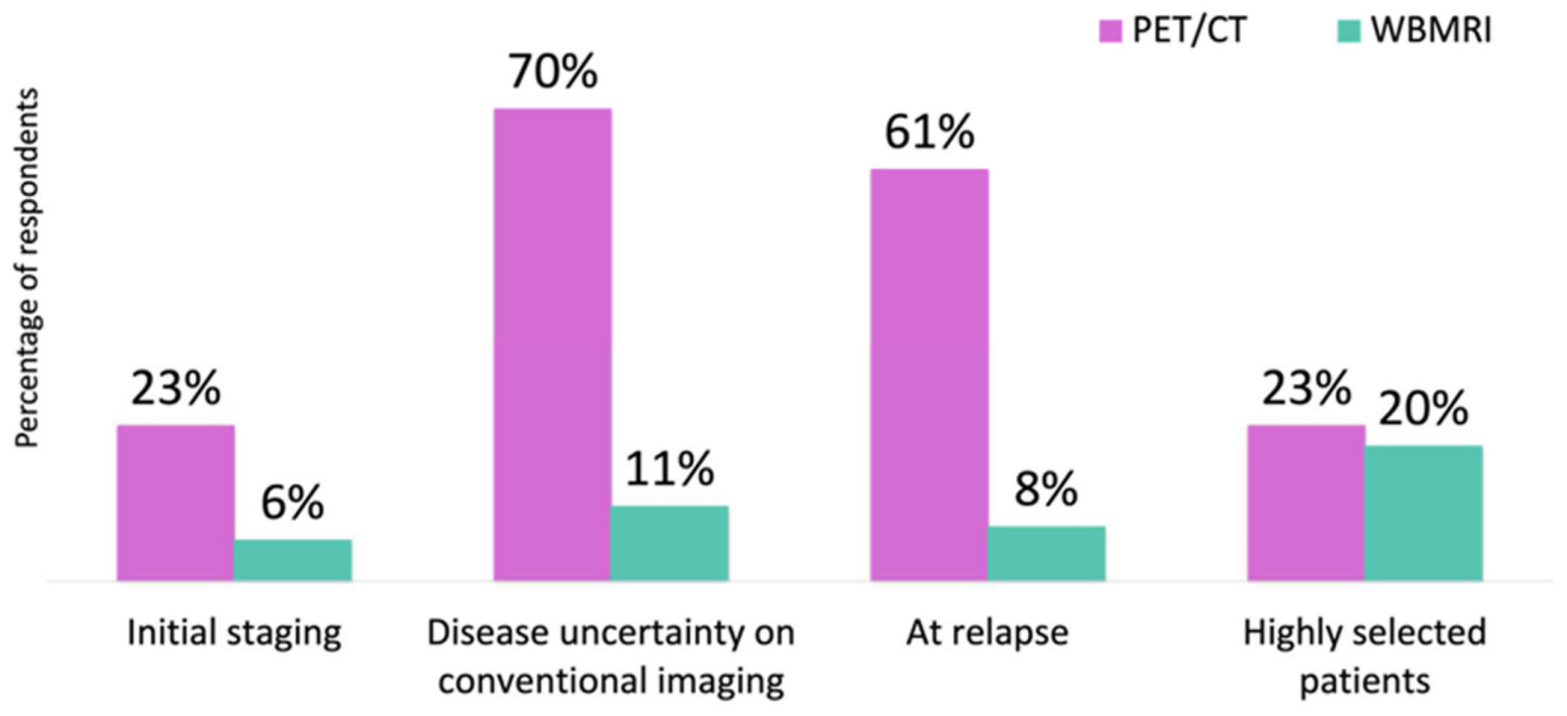
Timepoints for when clinicians request positron emission tomography/computerised tomography and whole-body magnetic resonance imaging.

In total, 24/64 (37.5%) respondents had access to whole-body magnetic resonance imaging (WBMRI) and 38/64 (59%) did not have access to WBMRI. The timepoints for when clinicians requested novel imaging with PET/CT or WBMRI scans are summarised in Figure 1.

**Fig 2:**
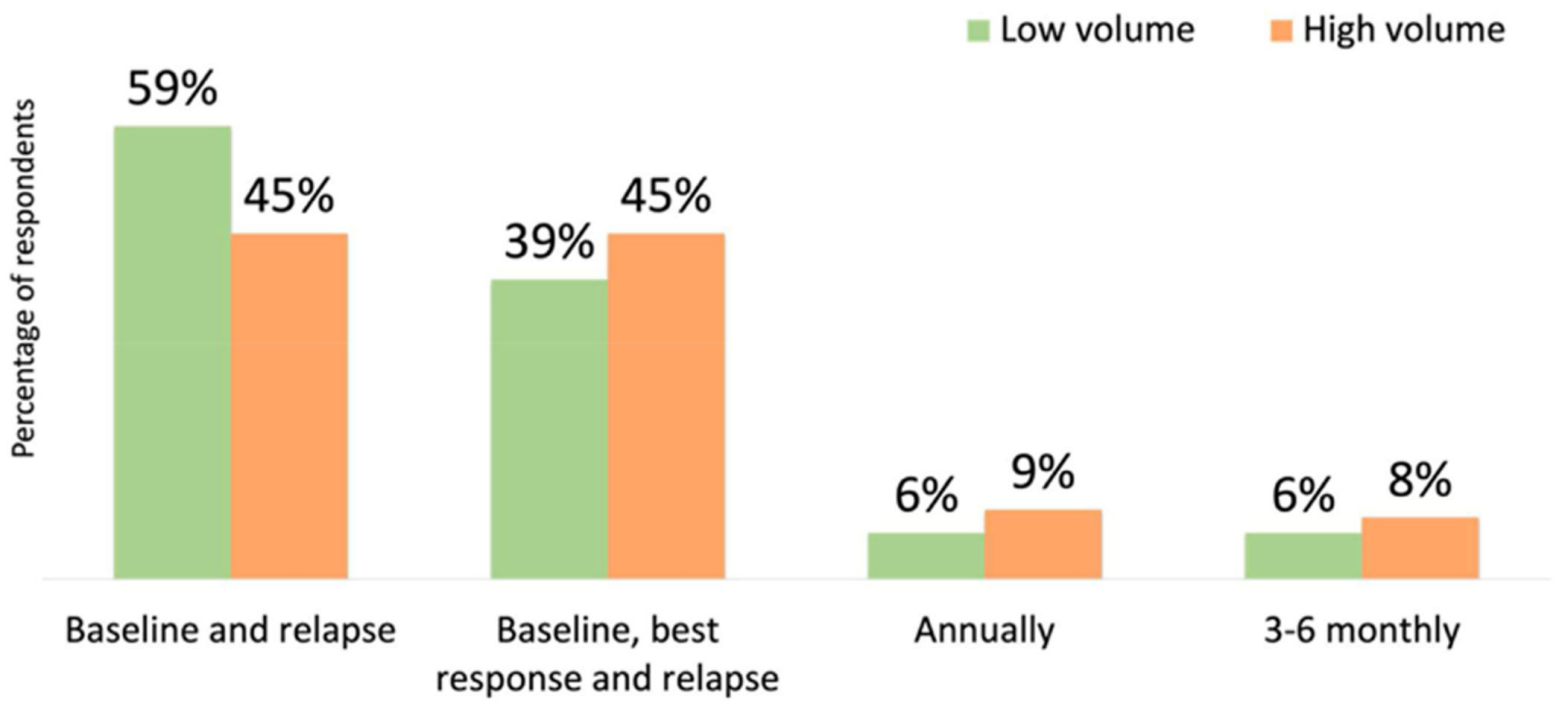
Frequency of imaging in low- and high-volume metastatic disease.

Questions on the frequency of imaging in mHSPC were divided based on disease volume (low volume versus high volume). For these questions, multiple responses were permitted. Figure 2 summarises the frequency of imaging in low- and high-volume mHSPC.

For best response assessment scans, 54/64 (84%) respondents selected CT and bone scans as the preferred imaging modality used, 3/64 (5%) respondents selected WBMRI and 2/64 (3%) respondents selected PET/CT.

**Fig 3:**
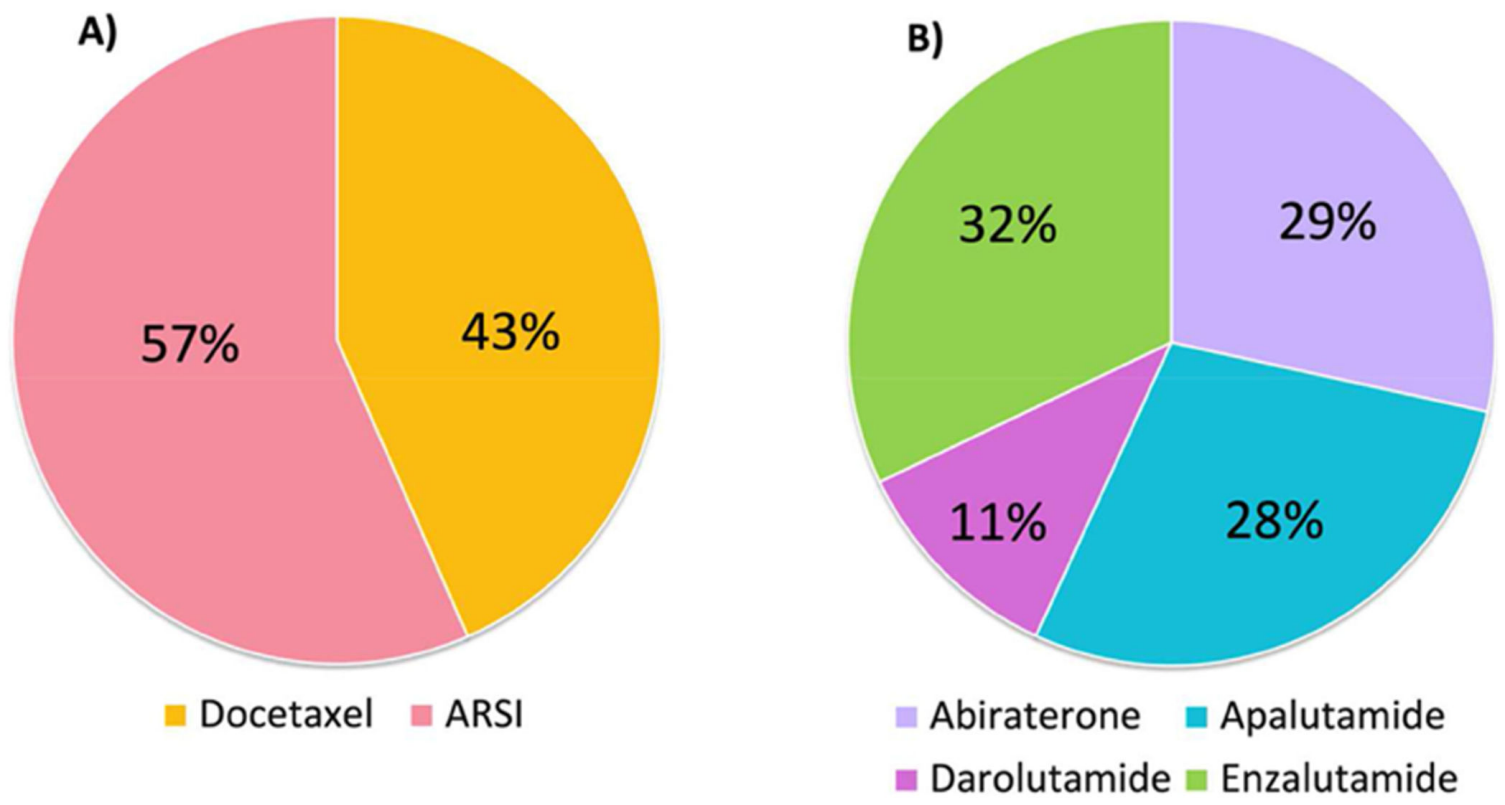
Systemic treatment. (A) Choice of doublet therapy with androgen deprivation therapy. (B) Choice of androgen receptor signalling inhibitors.

The choice of systemic doublet therapy in addition to ADT is summarised in Figure 3A. The choice of ARSI for systemic doublet therapy, providing all were approved and available on the National Health Service, is summarised in Figure 3B.

Forty-seven of 64 respondents (73%) were likely to start ARSI therapy together with ADT and 14/64 (22%) respondents were likely to start ARSI at any another time after starting ADT. Of those, 7/14 (50%) started ARSI within 3 months of ADT, 3/14 (21%) started within 6e8 weeks and 4/14 (29%) started within 1 month of ADT.

Twenty-nine of 64 respondents (45%) were likely to use docetaxel chemotherapy as part of triplet therapy, if funding was available, 10/64 (16%) were not likely to use triplet therapy and 22/64 (34%) were unsure.

**Fig 4:**
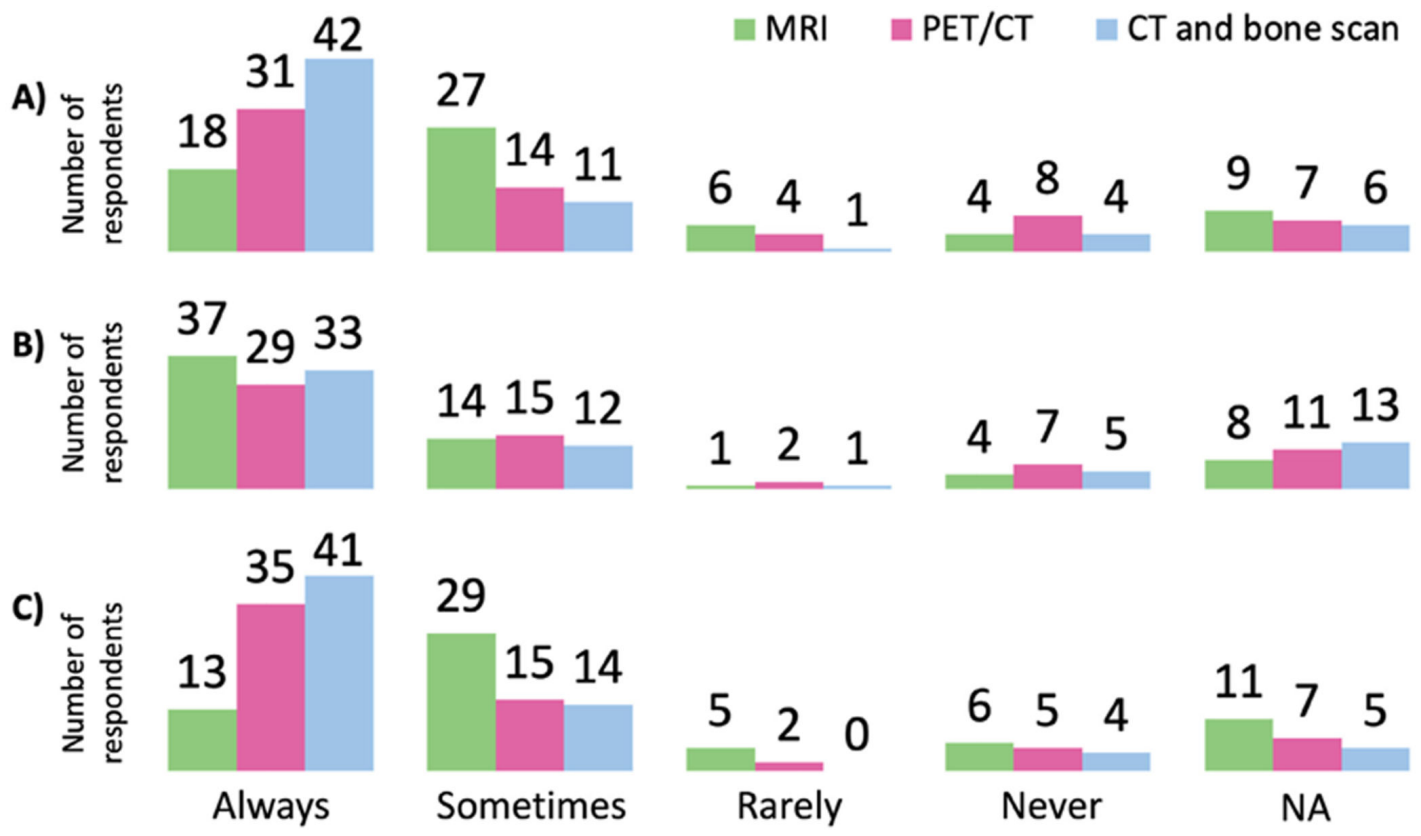
Frequency of each imaging modality requested for stereotactic ablative body radiotherapy planning to (A) bone (non-spinal), (B) spine and (C) lymph node metastases.

Forty-five of 64 respondents (70%) had access to SABR to treat spinal, non-spinal bone and nodal metastases at their treating centre and 22/64 (34%) had access through a neighbouring centre. For those who did not have direct access, 8/64 (12.5%) foresaw direct access at their treating centre in less than 1 year and 5/64 (8%) in 1–3 years. Figure 4 summarises the frequency of each imaging modality used for SABR planning for bone (non-spinal, Figure 4A), spine (Figure 4B) and lymph node metastases (Figure 4C).

For the delivery of prostate radiotherapy and SABR in comparison S, most respondents were participating sites in other National Institute for Health and Care Research portfolio prostate trials, this included 39/64 (61%) respondents who participated in the PACE umbrella trial (ISRCTN17627211), 37/64 (58%) in the PIVOTALboost trial (ISRCTN80146950) and 17/64 (26.5%) in the CORE trial (ISRCTN45961438). In oligometastatic disease, 48/64 (75%) respondents would treat lymph nodes if found to be involved on conventional imaging and 47/64 (73%) if involved on PET/CT.

Seven of 64 respondents (11%) had direct access to ^177^Lu-PSMA-617; 18/64 (28%) respondents had access through a neighbouring centre. For respondents with no direct access to ^177^Lu-PSMA-617, 16/64 (25%) foresaw access in less than 1 year, 18/64 (28%) foresaw access in 1–3 years, 1/64 (2%) foresaw access in more than 3 years, 8/64 (12.5%) had no access planned and 21/64 (33%) were unsure.

## Discussion

The STAMPEDE platform trial is a multi-arm multi-stage trial that has recruited 11 992 patients across 120 sites in the UK and Switzerland since its ethical approval in 2005. Responses to our survey were from UK-based participating sites only and have shown great interest for participation in the three new comparisons of the forthcoming STAMPEDE2 trial. Results from the survey have informed the final design of the STAMPEDE2 trial by concluding current practices in the UK related to access to novel imaging and choice of treatment in advanced prostate cancer.

We acknowledge that our survey received only a 48% response rate, which is moderate but not high and thus our findings cannot be regarded as fully representative of all STAMPEDE sites. The survey was conducted at a time of particular pressures within the National Health Service due to COVID-19, when there were limited staff for completion of the survey. One year on from this, the STAMPEDE2 trial is in set-up and enthusiasm from sites to take part in STAM-PEDE2 is evident, with a more responsive recent request eliciting strong interest to take part.

Our results showed the wide accessibility for PET/CT imaging. Most respondents requested PET/CT imaging at the time of disease relapse (61%) or disease uncertainty on conventional imaging (70%) given the greater accuracy of PET/CT for the detection of metastases when compared with suboptimal conventional imaging [20–22]. This practice reflects the established role of PET/CT imaging, in particular PSMA PET/CT in biochemically recurrent disease [23,24] and the initial staging of prostate cancer [22]. Results from these prospective trials have subsequently led to the endorsement of PSMA PET/CT imaging in updated international guidelines [7,8].

The improved sensitivity and specificity of PSMA PET/CT imaging for staging prostate cancer may redefine disease extent with potential stage migration and subsequent change in the patient’s treatment plan. Significant implications may arise from treatment alteration, leading to the omission of evidence-based treatment or overtreatment of what would otherwise be considered ‘microscopic’ disease. Evidence on clinical outcomes following PSMA PET/CT-directed treatment in mHSPC remains limited. In addition, current evidence from clinical trials for the management of prostate cancer is based on conventional imaging.

In non-metastatic prostate cancer, the survival benefit from combination treatment with ADT and radical doses of prostate radiotherapy is known [25,26]. Staging men in this group with PSMA PET/CT scans may detect occult metastatic disease resulting in the delivery of palliative doses of prostate radiotherapy or its omission. Similarly, the detection of low-volume metastatic disease may persuade the treating clinician to deliver SABR to metastatic sites with no real added benefit to men who will inevitably have excellent outcomes.

In low-burden metastatic disease detected on conventional imaging, the STAMPEDE M1:RT comparison demonstrated improved failure-free and overall survival with prostate radiotherapy [2,3]. Exploratory analysis showed that there was a continuum of benefit from prostate radiotherapy beyond three bone metastases seen on bone scans [27]. Additionally, bone scans were predictive of a response to prostate radiotherapy [28]. Oligometastatic disease has been defined as an intermediary metastatic state [29]. Its current definition is largely driven by the imaging modality used to describe the presence of a limited number of macroscopically visualised lesions [30–33]. In metachronous oligometastatic disease, SABR combined with standard of care improved progression-free survival [9–13]. A post-hoc analysis from the ORIOLE trial reported improved outcomes when all lesions visualised on PSMA PET/CT were treated with SABR [9].

WBMRI is a novel imaging modality with improved sensitivity than conventional imaging for bone metastases detection [34,35]. Standardised reporting guidelines have been published [36]. WBMRI can assess the cellularity of bone lesions and measure changes in apparent diffusion coefficient values, which has been correlated with treatment response [37–39]. The STAMPEDE2 trial comparison S eligibility will be determined by conventional imaging as per current clinical evidence. Considering the current status quo with access to novel imaging, an imaging sub-study will be integrated in STAMPEDE2 comparison S and treatment decisions using novel imaging will be stratified (see Supplementary Appendix B). The sub-study aims to explore patterns of treatment decisions and clinical outcomes for each imaging modality in the context of a large prospective clinical trial.

The survey concluded that almost half of clinicians (43%) used docetaxel as doublet therapy, despite National Institute for Clinical Excellence approval for enzalutamide and apalutamide in mHSPC following the COVID-19 pandemic [40,41]. Results from the STAMPEDE, CHAARTED and LATI-TUDE trials have shown that the addition of abiraterone or docetaxel to long-term ADT improves survival [4–6,42,43]. However, no trials have directly compared the two treatments to determine superiority of one over the other. A post-hoc analysis from the STAMPEDE trial compared outcomes from the abiraterone and docetaxel contemporaneous comparisons where recruitment overlapped, the results of which favoured abiraterone for improved failure-free survival and progression-free survival, with no significant difference with regards to other outcomes [44]. Subsequent exploratory analysis from the STAMPEDE trial reported on quality of life differences between the two treatments. The results after 2 years of treatment showed an improved global quality of life score with abiraterone [45]. In STAMPEDE2, based on our own patient-reported outcome data [46], we have adopted ARSI as the doublet treatment of choice. The choice of ARSI doublet treatment aligns with the investigational treatment in the trial. Additionally, by offering biomarker testing prior to starting ARSI, there is the opportunity for a second randomisation in comparison N for patients with a positive biomarker status (see Supplementary Appendix C).

Triplet therapy was likely to be used by 45% of respondents. At the time of the survey, the PEACE-1 and ARASENS trials had reported on the improved overall and progression-free survival with triplet therapy [47,48]. Subgroup analysis from the ARASENS trial confirmed that the survival benefit was consistent among all comers regardless of the disease volume and risk, with no increased toxicity from the addition of ARSI [49]. Since the survey, darolutamide has recently become available on the National Health Service for men with mHSPC through the ‘fast-track access for life-extending drugs’ scheme [50]. In the STAM-PEDE2 trial design, provision has been made for triplet therapy use across all comparisons. The decision to treat with triplet therapy will be stratified and will be at the treating clinician’s discretion.

Best response assessment scans may be useful when the clinical trial primary endpoint is radiographic progression as per the RECISTv1.1 criteria [51]. In the STAMPEDE2 trial, we strongly recommend undertaking best response assessment scans at 24 weeks from randomisation to correspond with the prostate-specific antigen nadir. The preferred choice of scans would be CT and bone scans to facilitate a validated like-for-like comparison with baseline and progression scans.

Radiotherapy quality assurance for the STAMPEDE2 trial comparison S will be led by the national Radiotherapy Clinical Trials Quality Assurance group and will be streamlined through the SABR expansion programme and other National Institute for Health and Care Research portfolio prostate cancer trials (PACE: ISRCTN17627211, PIVOTALboost: ISRCTN80146950 and PEARLS: ISRCTN36344989 trials). The survey results have shown that most centres had access to SABR. We, therefore, anticipate a smooth set-up and start to recruitment in comparison S.

Following the Food and Drug Administration approval for ^177^Lu-PSMA-617 in CRPC [16], ^177^Lu-PSMA-617 became available in the UK through the Early Access to Medicines Scheme [52], potentially expanding access across the UK. At the time of writing, the therapy is no longer available pending a National Institute for Clinical Excellence review. Treating centres with an infrastructure to support radioactive ligand therapy delivery will be prioritised to open for recruitment in the STAMPEDE2 trial comparison P. Additionally, this comparison is part sponsored by Advanced Accelerator Applications USA, Inc (AAA, a Novartis company; Millburn, NJ, USA) who will supply ^177^Lu-PSMA-617 and support additional trial costs.

## Conclusion

The STAMPEDE2 trial will open three new investigative treatment comparisons for men diagnosed with mHSPC. There is significant variation in clinical practice across current study sites regarding access and application of novel imaging and the choice of therapy combinations at treatment initiation. Despite this, most existing trial centres have expressed great interest in participation in the STAMPEDE2 trial.

## Supplementary Material

Supplementary data

## Figures and Tables

**Table 1:** Type of available positron emission tomography/computerised tomography (PET/CT) imaging

Access to PET/CT imaging	*n*/*N*	% (95% confidence interval)
PET/CT	62/64	97 (89–99)
^18^F-choline PET/CT	36/62	58 (45–70)
^18^F-PSMA PET/CT	25/62	40 (28–54)
^68^Ga-PSMA PET/CT	23/62	37 (25–50)

F, fluorinated; Ga, gallium; PSMA, prostate-specific membrane antigen.
