# Supplementary material for: The STAMPEDE2 Trial: a Site Survey of Current Patterns of Care, Access to Imaging and Treatment of Metastatic Prostate Cancer
Source: Clin Oncol (R Coll Radiol). Author manuscript; Available in PMC 2024 Nov 16. (PMC7616818; doi:10.1016/j.clon.2023.07.009)

# Appendices

## Appendix A- STAMPEDE2 site survey

STAMPEDE2 SITE SURVEY

We would be very grateful if you could take some time to complete this survey for the development of STAMPEDE-2 trial platform. It consists of 6 parts and will take approximately 10 minutes.

**PART 1 – General questions**

1. Please provide which site you are based at e.g., Somewhere General Hospital
2. Please provide your job title
3. Please provide your name (optional)
4. Would you participate in a comparison that randomises oligometastatic patients to SABR vs no SABR in addition to SOC?
   - - Yes
     - No
     - Unsure
     - If No or Unsure, please explain in the comments box
5. Would you participate in a comparison that randomises polymetastatic patients to PSMA Lutetium-617 vs no PSA Lutetium-617 in addition to SOC.?
   - - Yes
     - No
     - Unsure
     - If No or Unsure, please explain in the comments box
6. Would you participate in a comparison that randomises DDR positive patients to Abiraterone and Niraparib vs SOC?
   - - Yes
     - No
     - Unsure
     - If No or Unsure, please explain in the comments box

**Part 2- Questions relating to imaging facilities at your site**

1. Do you have access to PET/CT imaging?

- Yes, PET/CT is available at my centre
- Yes, PET/CT is available at a neighbouring centre
- Yes, PET/CT is available at a distant centre with a long referral pathway
- No

1. What type of PET/CT imaging do you use currently (use numbers or vague estimates for proportion if more than one)?

- Choline PET/CT
- 68-Gallium-11 PSMA PET/CT
- 18-Fluoride PSMA PET/CT
- Other, please specify

1. If you currently have access to PET/CT, when are you likely to request this for your patient?

- At the Initial staging
- If there is disease uncertainty on conventional imaging
- At time of relapse
- In highly selected patients, please specify

1. If you don’t have access to PET/CT imaging, do you foresee this happening in

- less than 1 year
- 1-3 years
- >3 years
- No access planned
- Unsure (please explain in comments)

1. For non- spinal bone lesions in SABR planning, which imaging modality do you use and at what frequency (you may select multiple responses)?

- MRI
  - Always
  - Sometimes
  - Rarely
  - Never
- PET/CT
  - Always
  - Sometimes
  - Rarely
  - Never
- CT and bone scan
  - Always
  - Sometimes
  - Rarely
  - Never
- Other modalities, please specify

1. For spinal bone lesions in SABR planning, which imaging modality do you use and at what frequency (you may select multiple responses)?

- MRI
  - Always
  - Sometimes
  - Rarely
  - Never
- PET/CT
  - Always
  - Sometimes
  - Rarely
  - Never
- CT and bone scan
  - Always
  - Sometimes
  - Rarely
  - Never
- Other modalities, please specify in comments box

1. For lymph node lesions in SABR planning, which imaging modality do you use and at what frequency (you may select multiple responses)?

- MRI
  - Always
  - Sometimes
  - Rarely
  - Never
- PET/CT
  - Always
  - Sometimes
  - Rarely
  - Never
- CT and bone scan
  - Always
  - Sometimes
  - Rarely
  - Never
- Other modalities, please specify in comments box

1. Do you have access to Whole body MRI imaging?

- Y/N

1. If you currently have access to Whole body MRI imaging, when are you likely to request this for your patient?

- At the Initial staging
- If there is disease uncertainty on conventional imaging
- At time of relapse
- In highly selected patients, please specify

1. How often do you currently scan as standard of care in men with low volume mHSPC? (You may select multiple responses)

- At baseline (pre-ADT) and PSA/clinical progression only
- At baseline, best response (corresponding to PSA nadir), and PSA/clinical progression
- On a yearly basis
- At regular time intervals, please specify
- Other, please specify

1. How often do you currently scan as standard of care in men with high volume mHSPC? (You may select multiple responses)

- At baseline (pre-ADT) and PSA/clinical relapse only
- At baseline, best response (corresponding to PSA nadir), and PSA/clinical progression
- On a yearly basis
- At regular time intervals, please specify
- Other, please specify

1. What imaging modality do you use to assess best response to treatment?

- MRI
- PET/CT
- CT and Bone scan
- Other, please specify in comments box

**Part 3- Questions relating to your use of systemic therapies**

1. In addition to ADT, which systemic treatment are you currently using for metastatic HSPC? You can tick both.

- ARSi
- Docetaxel

1. If funded, are you likely to use docetaxel in addition to ADT and ARSi, as part of triple therapy?

- Y/N
- Unsure
- Please specify in the comments box reasoning for your response

1. The trial will require ADT + one ARSi. If all were approved, what would be your ARSi of choice? (Please use vague estimates for proportion if more than one)

- Abiraterone
- Apalutamide
- Enzalutamide
- Darolutamide

1. When are you likely to start ARSi therapy in metastatic patients?

- At the time of commencing ADT
- At the time of radiotherapy/PSMA Lu
- Any other time, please specify

**Part 4- Questions relating to the SABR comparison**

1. Do you have access to SABR at your centre to treat bone, lymph nodes and spinal metastases?

- Y/N (if Y, please specify which of the above sites of disease)

1. If no to question 23 (SABR access), do you foresee this happening in

- less than 1 year
- 1-3 years
- >3 years
- Not planned
- Unsure (please explain in comments box)

1. If no to question 23, is there a neighbouring centre that you currently refer to for SABR delivery?

- Y/N (if Y, please specify referral site)

1. If you don’t have RTTQA approval for SABR, are you prepared to get benchmark approval to deliver SABR to bone, lymph nodes and spinal metastases?

- Y/N

1. Was/Is your site participating in the following trials (you may select multiple responses)?

- CORE trial
- PACE trial
- PIVOTAL Boost trial
- None

1. In oligometastatic disease, do you plan to treat pelvic lymph nodes with radiotherapy?
2. If LN involved on conventional imaging:

- Y/N

1. If LN not involved on conventional imaging:

- Y/N

1. If LN involved on PSMA PET/CT

- Y/N

1. If LN not involved on PSMA PET/CT

- Y/N

1. We recommend a moderately hypofractionated RT schedule for pelvic LN RT (60Gy in 20#, 47Gy in 20# to LN), do you plan to use this dose fractionation?

- Y/N
- If N, please specify why and describe your preferred fractionation schedule

**Part 5- Questions relating to the 177Lu-PSMA-617 comparison**

1. Do you currently have access to 177Lu-PSMA-617 treatment at your centre?
   1. Y/N
2. If you currently don’t have access to 177Lu-PSMA-617 at your centre, is there a neighbouring centre that you could refer to?

- Y/N
- if Y please specify referral site
- NA

1. If you currently don’t have access to 177Lu-PSMA-617 at your centre, do you foresee this happening in

- less than 1 year
- 1-3 years
- >3 years
- No access planned
- Unsure (please explain in comments)

1. **Please feel free to provide any other comments in the box below:**

**Thank you for taking the time to complete this survey.**

## Appendix B- Comparison S imaging sub-study flowchart


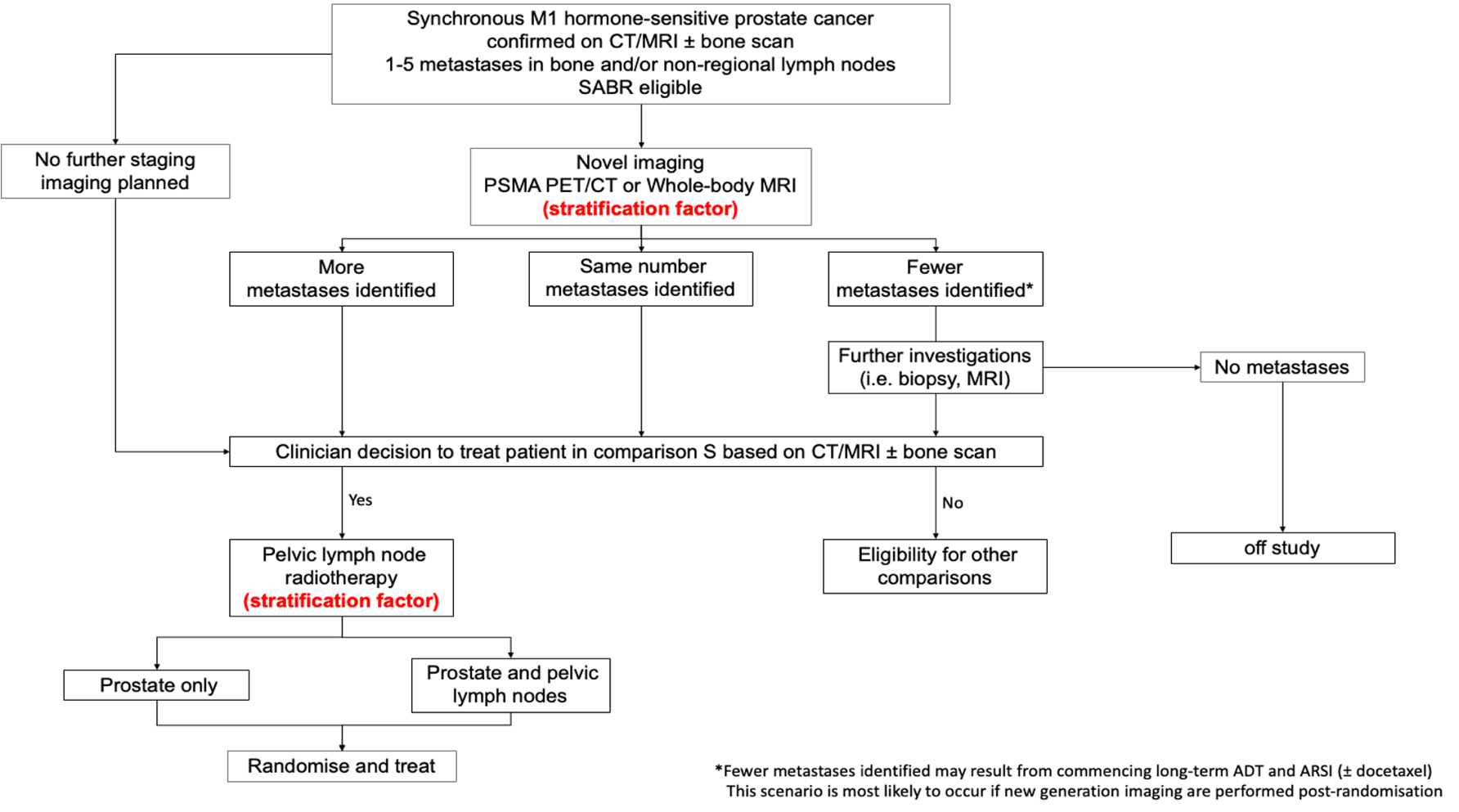
Comparison S imaging flowchart. Integration of novel imaging sub-study with PSMA PET/CT and WBMRI. Treatment decisions will be stratified based on the imaging modality used. This applies to treatment decision for radiotherapy treatment to pelvic lymph nodes.

## Appendix C- STAMPEDE2 trial schema

STAMPEDE2 trial schema. Two-by-two co-enrolment design. Confirmation of eligibility based on conventional imaging (CT/MRI and bone scan). Upfront radiation-based randomisation dependent on SABR eligibility as specified in the protocol. Randomisation in comparison S if SABR eligible and comparison P if SABR ineligible. Upfront biomarker status testing for men who have not commenced on ARSI. For biomarker status positive patients, they will be offered a second biomarker randomisation in comparison N to abiraterone plus niraparib.


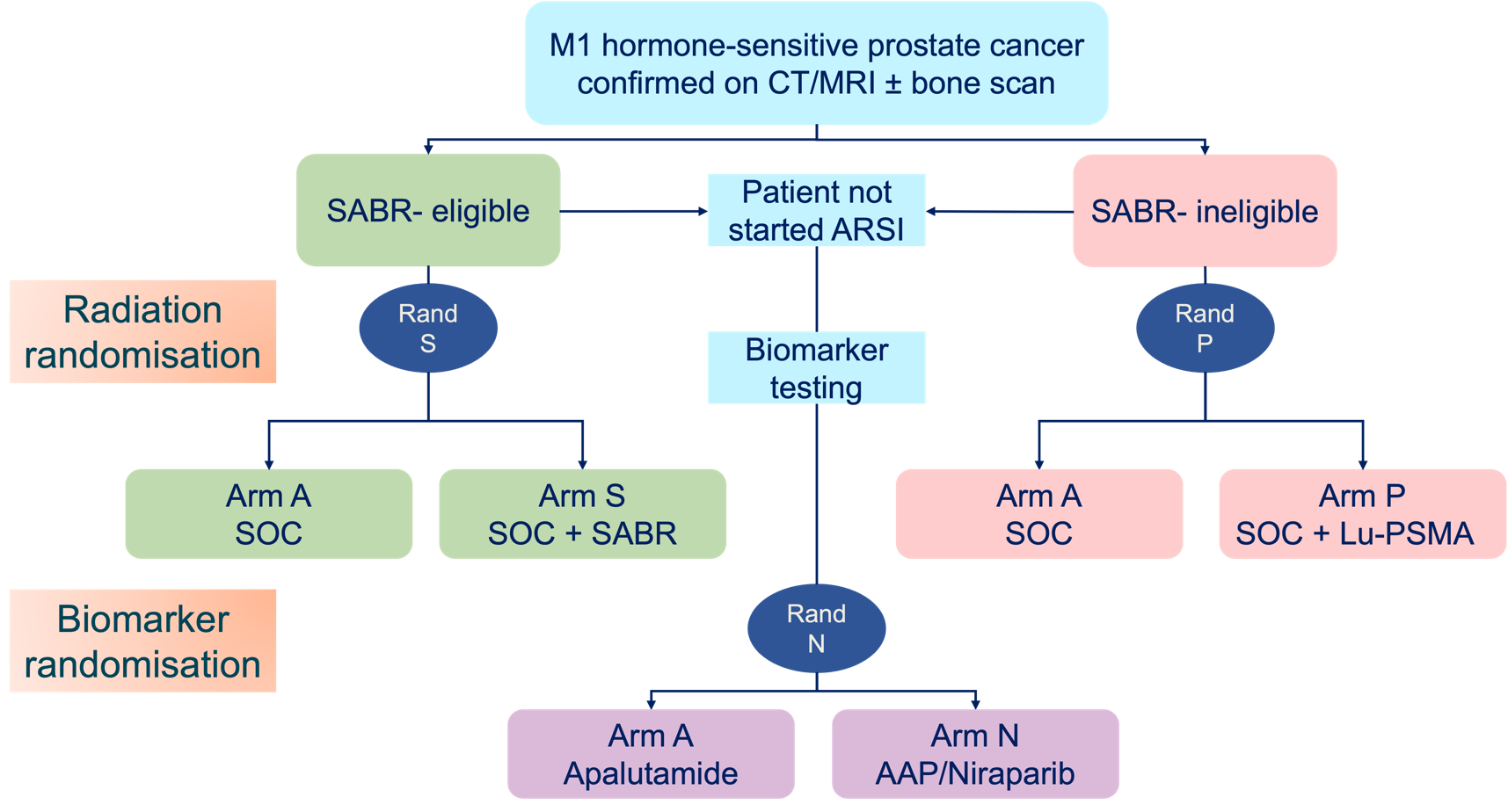

Supplement: Supplementary data [file EMS200033-supplement-Supplementary_data.docx]
